# Recent advances on the synthesis of natural pyrrolizidine alkaloids: alexine, and its stereoisomers

**DOI:** 10.1007/s13659-022-00324-5

**Published:** 2022-02-07

**Authors:** Ghodsi Mohammadi Ziarani, Negar Jamasbi, Fatemeh Mohajer

**Affiliations:** Department of Chemistry, Faculty of Physics and Chemistry, Alzahra University, P. O. Box 1993893973, Tehran, Iran

**Keywords:** Polyhydroxylated pyrrolizidine alkaloids, Alexine, Natural products, Alexa leiopetala

## Abstract

Natural products have attracted the interest of the scientific community due to their importance and application. Alexine is a naturally polyhydroxylated pyrrolizidine alkaloid that is broadly found in plant sources and isolated from *Alexa leiopetala*. The biological properties such as glycosidase inhibitors, anti-virus, and anti-HIV activities, makes it interesting target for synthetical studies. This review reports different approaches and methodologies to the synthesis of alexine, and its stereoisomers as the target compounds in numerous studies.

**Graphical Abstract:**

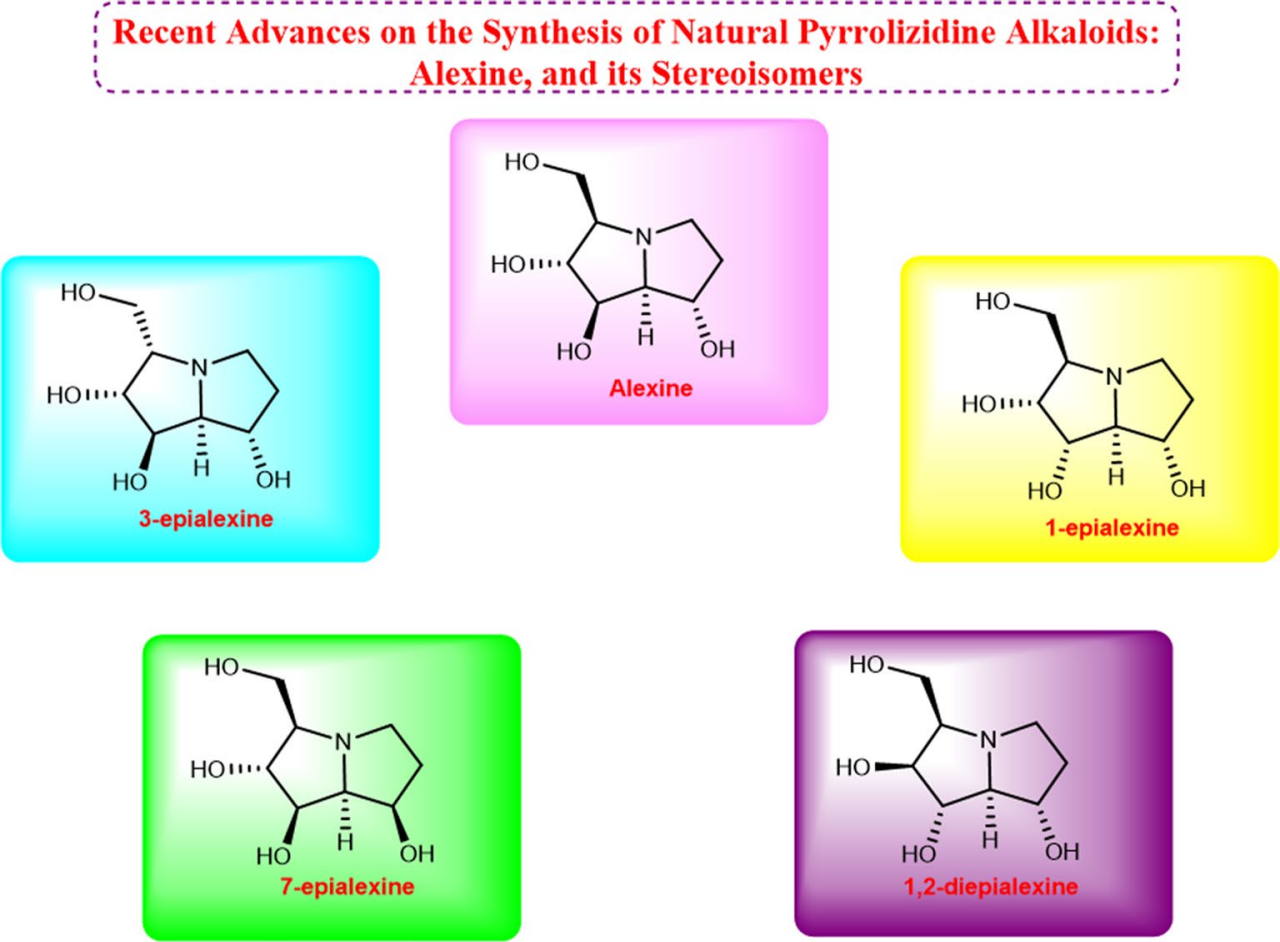

## Introduction

**Fig. 1:**
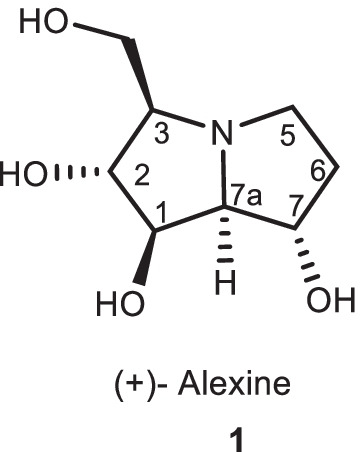
The structures of alexine **1**

Pyrrolizidine alkaloids are in numerous active natural compounds and isolated mainly from plants of the Asteraceae, Boraginaceae, Fabaceae, and Orchidaceae families [1]. Alexine **1** is a naturally polyhydroxylated pyrrolizidine alkaloid, which was functionalized by the hydroxyl stereocenter groups (Fig. 1). This compound is a class of sugar mimics with the l-*aza-*bicyclo-[3.3.0]-octane skeleton that they are isolated from plants [2–4].

In 1988, Alexine **1** was isolated from *Alexa leiopetala* by Nash et al. and its structure and the absolute configuration was revelead using X-ray crystallography [5]. Although many pyrrolizidine alkaloids were previously isolated having carbon substituents at C-1, alexine **1** was the first example of a pyrrolizidine alkaloid with a C-3 hydroxymethyl branch [6].

**Fig. 2:**
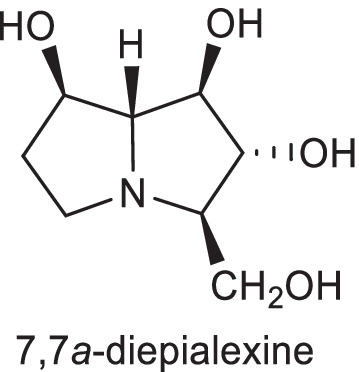
The structure of 7,7*a*-diepialexine

The potential of these compounds as selective glycosidase inhibitors, as well as their antiviral and retroviral activities, have received much attention from many researchers [7–10]. For example, 7,7*a*-diepialexine is one of the useful compounds, which exhibit antiviral effects against HIV virus growth by reducing the cleavage of precursor HIV-1 glycoprotein 160 (gp160) (Fig. 2) [9].

Due to the biological activities and stereochemically hydroxyl groups in the pyrrolizidine skeleton, alexine **1** is an important target in organic synthesis. So far, many research groups have effort to synthesis this type of pyrrolizidine alkaloids, and several review articles have been published [11, 12]. Therefore, in continuation of our previous studies about naturally occurring alkaloids [13–21], the synthesis of the natural pyrrolizidine alexine **1** and its stereoisomers were reviewed from 1988 till now with different strategies and methodologies for the synthesis of these compounds.

## Total synthesis of natural pyrrolizidine alkaloids: alexine, and its Stereoisomers

### The synthetic approach of Fleet and co-workers

**Scheme 1:**
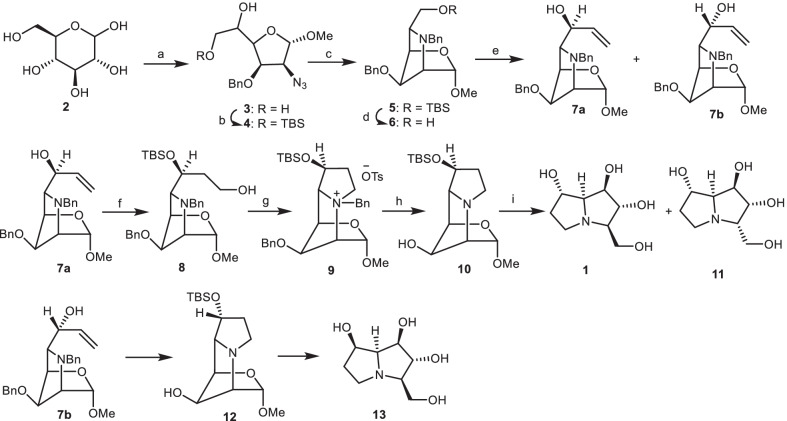
Fleet method for the synthesis of the alexine **1**, 3-epialexine **11,** and 7-epialexine **13**. Reagents and conditions: (a) ref [23]; (b) TBSCl, DMF, 0 °C, 95%; (c) (i)Tf_2_O, pyridine, − 30 °C; (ii) H_2_, Pd, EtOAc; (iii) BnBr, DMF, NaOH, 77% overall for 3 steps; (d) TBAF, THF, 88%; (e) (i) oxalyl chloride, DMSO, Et_3_N; (ii) CH_2_$=$CHMgBr, THF, **7a**: 38%, **7b**: 37%; (f) (i) TBSCl, 89%; (ii) BH_3_.DMS, THF, then H_2_O_2_, OH, 67%; (g) (i) *p*-TsCl, pyridine, CH_2_Cl_2_, 77%; (h) H_2_, Pd/C, aq. acetic acid, 72%; (i) (i) TFA/H_2_O, 36 h; (ii) NaBH_4_, EtOH, 54%.

In 1988, Fleet et al. [22] reported the synthesis of alexine **1**, 3-epialexine **11**, and 7-epialexine **13** from *α-d*-mannofuranoside derivative **3**, which was prepared from d-glucose **2** [23]. The diol **3** was reacted with *tert*-butyldimethylsilyl chloride (TBSCl) to obtain the compound **4**, which was esterified with trifluoromethane sulphonic anhydride (Tf_2_O), followed by hydrogenation over H_2_/Pd and reaction with benzyl bromide (BnBr) to give the fully protected pyrrolidine **5** in three steps. Deprotection of TBS group through the reaction of resulting product **5** with tetrabutylammonium fluoride (TBAF) provided the compound **6**, which was submitted to Swern oxidation [24] [oxalyl chloride (COCl)_2_/dimethyl sulphoxide (DMSO), triethylamine (Et_3_N)], followed by reaction with vinyl magnesium bromide (CH_2_=CHMgBr) to give the epimeric allylic alcohols **7a** and **7b**. The allylic alcohol **7a** was reacted with TBSCl to produce the silyl ether, which was treated with borane dimethylsulfide through the hydroboration reaction, then the resulting silyl ether reacted with alkaline hydrogen peroxide to result in the primary alcohol **9**. Then, it was esterified with *p*-toluenesulphonyl chloride (*p*-TsCl) to obtain a tosylate, which was cyclized to give the salt **9**. Hydrogenolysis of the latter by H_2_, Pd/C gave the tricyclic compound **10**, which was treated with trifluoroacetic acid (TFA), followed by the reduction with NaBH_4_ to yield the alexine **1**. A small amount of 3-epialexine **11** was also obtained and presumably arose from the epimerization of the open-chain form of the intermediate lactol. On the other hand, another alcohol **7b** was converted into 7-epialexine **13** by a similar synthetic process (Scheme 1).

**Scheme 2:**
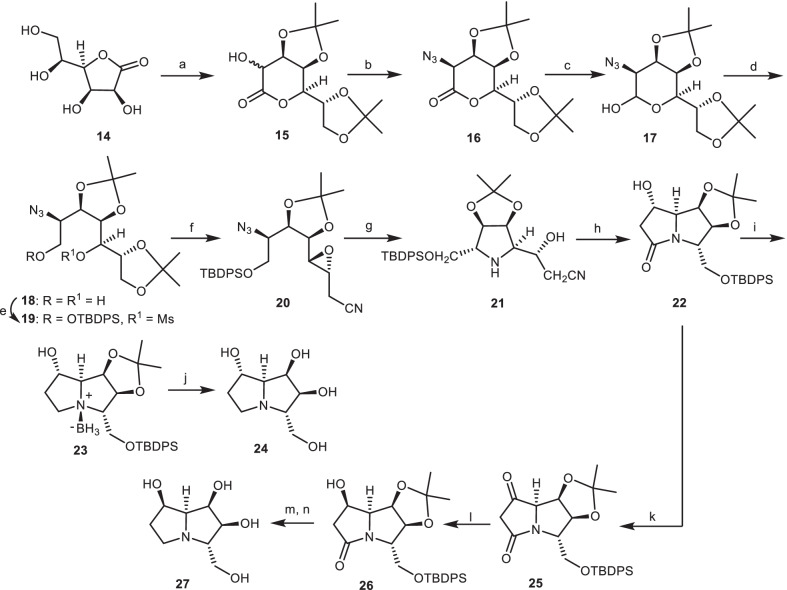
The synthesis of the 1,7,7*a*-triepialexine **24** and 1,7*a*-diepialexme **27** by Fleet et al. Reagents and conditions: (a) ref [26]; (b) (i) Tf_2_O, 85%; (ii) NaN_3_ in DMF, r.t., 90%; (c) DIBALH in THF; (d) NaBH_4_ in MeOH; (e) TBDPSCl, imidazole, DMF, 0 °C, then MsCl, DMAP in pyridine; (f) (i) HOAc in aq. 1,4-dioxan; (ii) Ba(OMe)_2_ in MeOH, 0 °C; (iii) Tf_2_O in CH_2_Cl_2_, − 50 °C; (iv) LiCN in CH_2_Cl_2_/ THF; (g) H_2_, Pd black, EtOAc; (h) NH_4_Cl, 35% aq. NH_3_-EtOH, 100 °C, 20 h; (i) THF.Borane complex in THF; (j) 50% aq.CF_3_COOH; (k) PCC, CH_2_Cl_2_; (l) NaBH_4_, EtOH, 0 °C; (m) THF.Borane complex in THF; (n) 50% aq.CF_3_COOH.

In another study, Fleet et al. investigated the application of the acetonide of heptonolactone **15** as starting material with additional chiral centers, which was derived from l-gluconolactone **14** for the synthesis of 1,7,7*a*-triepialexine **24** and 1,7*a*-diepialexine **27** [25, 26]. The acetonide of heptonolactone **15** was converted to azide **16** through the reaction with sodium azide (NaN_3_). Reduction of azide **16** with diisobutylaluminum hydride (DIBALH) provided the lactol **17**, which was reduced by NaBH_4_ to give diol **18**. Then, the diol **18** was protected and mesylated to obtain the mesylate **19**. Through the removal of terminal acetonide moiety of mesylate **19**, followed by the epoxidation with barium methoxide [Ba(OMe)_2_], the esterification of the remaining primary alcohol as its triflate, and subsequent treatment with LiCN, the epoxy-nitrile **20** was obtained in four steps. Through the hydrogenolysis of the epoxy-nitrile **20** with H_2_/Pd black, the key intermediate **21** was obtained. Then, the key intermediate **21** was reacted with ammonium chloride (NH_4_Cl) to give the bicyclic lactam **22**, which was reduced to the amine-borane adduct **23**. By the reaction of compound **23** with trifluoroacetic acid (CF_3_COOH), the target compound 1,7,7*a*-triepialexine **24** was obtained. The bicyclic lactam **22** was oxidized to ketone **25**, which was reduced to the alcohol **26**. Then, it was converted to 1,7*a*-diepialexine **27** through the similar synthetic process, which provided the compound of 1,7,7*a*-triepialexine **24** (Scheme 2).

### The synthetic approach of Ikota and co-workers

**Scheme 3:**
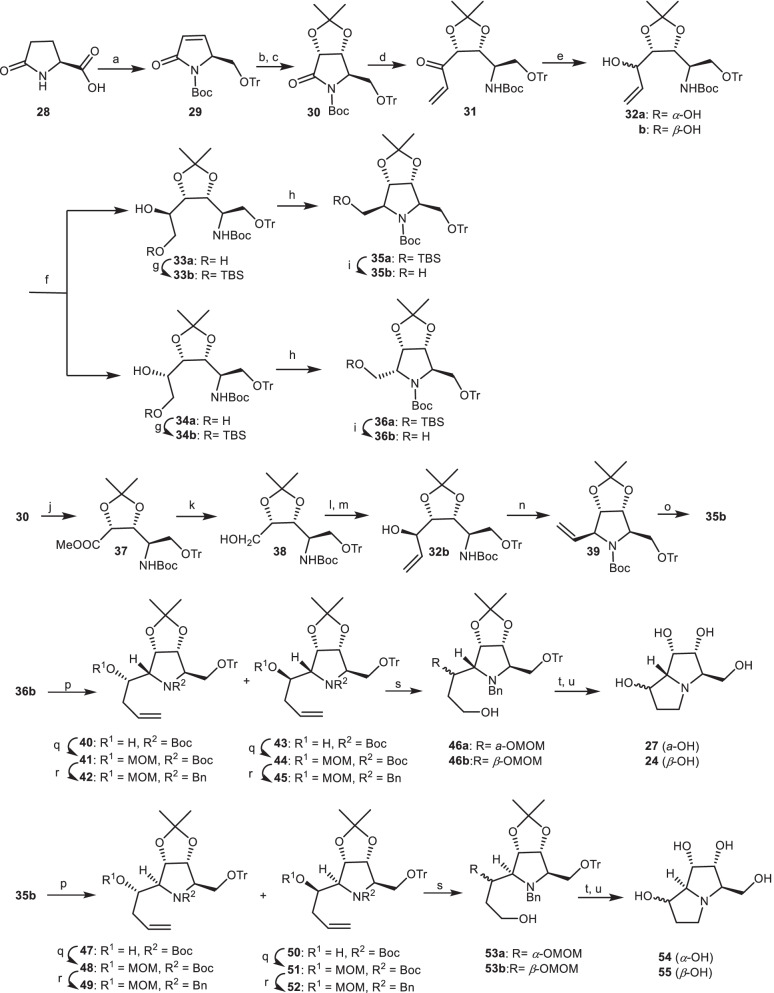
The synthesis of the 1-epialexine **54**, 1,7-diepialexine **55**, 1,7*a*-diepialexine **27**, and 1,7,7*a*-triepialexine **24** by Ikota and his co-workers. Reagents and conditions: (a) ref [28]; (b) OsO_4_, NMO, acetone/H_2_O, r.t., 20 h, 88%; (c) (CH_3_)_2_C(OCH_3_)_2_, *p-*TsOH, acetone, r.t., 2 h, 88%; (d) CH_2_=CHMgBr, THF, − 40 °C, 3 h, 93%; (e) NaBH_4_, CeCl_3_.7H_2_O, MeOH, 0 °C to r.t., 10 min to 1 h, 91%; (f) (i) O_3_, CH_2_Cl_2_, -78 °C, 5 min; (ii) NaBH_4_, EtOH, 0 °C, 15 min; (g) TBSCl, imidazole, DMF, 0 °C, **33b** (48%), **34b** (19%); (h) (i) MsC1, Et_3_N, CH_2_Cl_2_; (ii) *tert*-BuOK, THF, **35a** (75%), **36a** (78%); (i) TBAF, THF, r.t., **35b** (78%), **36b** (82%); (j) LiOH (aq.), THF/MeOH, then CH_2_N_2_, ether; (k) NaBH_4_, EtOH, 79%; (1) Swern oxidation, − 20 °C; (m) CH_2_=CHMgBr, − 78 °C, 71%; (n) (i) MsC1, Et_3_N, CH_2_Cl_2_; (ii) *tert*-BuOK, THF; (o) (i) O_3_, CH_2_Cl_2_, -78 °C; (ii) NaBH_4_, EtOH, 72%; (p) (i) Swern oxidation, − 78 °C to 20 °C, 10 min; (ii) CH_2_=CHCH_2_Li, − 78 °C, 1 h; (q) MOMCl, *N*,*N*-diethylaniline, CH_2_Cl_2_, r.t., 40 h; (r) (i) TBSOTf, 2,6-lutidine, CH_2_Cl_2_, r.t., 3 h; (ii) TBAF, THF, (iii) BnBr, K_2_CO_3_, acetone, r.t., 2 h; (s) O_3_, CH_2_Cl_2_, − 78 °C, then NaBH_4_, EtOH, 15 min; (t) MsCl, Et_3_N, CH_2_Cl_2_, r.t., 16 h, then 10% Pd/C, H_2_, HCl/EtOH, r.t., 1 h; (u) 10% HCl/MeOH, 70 °C, **27** (52%), **24**, **54**, **55** (13–21%).

Ikota and his co-workers applied optically active pyroglutamic acid derivatives for the natural product synthesis [27–29]. The stereocontrolled synthesis of 1-epialexine **54**, 1,7-diepialexine **55**, 1,7,7*a*-triepialexine **24** and 1,7*a*-diepialexine **27** were reported based on nonecarbohydrate through (*S*)-pyroglutamic acid derivative **30** [30, 31]. Dihydroxylation of the unsaturated lactam **29** in the presence of OsO_4_ as catalyst, followed by isopropylidenation to provide dioxolo derivative **30**. The addition of vinylmagnesium bromide (CH_2_=CHMgBr) to dioxolo derivative **30** through the Grignard reaction provided the *α,β*-unsaturated ketone **31**, which was reduced to diastereometic allylic alcohol **32**. Through ozonolysis process of the allylic alcohol **32**, followed by the reduction afforded a mixture of diols, which were transformed to the protected ethers **33b** and **34b**. The resulting ethers were mesylated by methanesulfonyl chloride (MsCl) followed by cyclization in the presence of *tert*-BuOK to give the protected pyrrolidines **35a** and **36a**, which were deprotected by *tert*-butylammonium fluoride (TBAF) to give the alcohols **35b** and **36b**. On the other hand, **35b** also was prepared in an alternative pathway through the removal of lactam moiety of **30**, followed by esterification using CH_2_N_2_ to provide methyl ester **37**. Then, it was reduced to alcohol **38**, which was oxidized by Swern reagent followed by treatment with vinylmagnesium bromide to afford the allylic alcohol **32b**. The alcohol **32b** was mesylated and cyclized to give the 5-vinylpyrrolidine **39**. Then, the 5-vinylpyrrolidine **39** converted to pyrrolidine **35b** via ozonolysis and subsequent reduction. Swern oxidation of the alcohol **36b**, and subsequent reaction with allyl magnesium chloride provided allylic alcohols **40** and **43**, which were protected by MOMCl to their methoxymethyl (MOM) ethers, followed by the conversion of *N-tert*-butyloxycarbonyl (*N*-Boc) group into the *N*-Benzyl (*N*-Bn) protecting group to give the epimeric mixture of **42** and **45**. Then, they were ozonolized and reduced to the alcohol **46**, which was mesylated and cyclized to the pyrrolizidine skeleton, followed by hydrogenolysis, and then their acidic treatment gave 1,7*a*-diepialexine **27** and 1,7,7*a*-triepialexine **24**. Through a similar sequence and synthetic pathways, 1-epialexine **54** (*α*-OH) and 1,7-diepialexine **55** (*β*-OH) produced using **35b** (Scheme 3).

### The synthetic approach of Yoda and co-workers

**Scheme 4:**
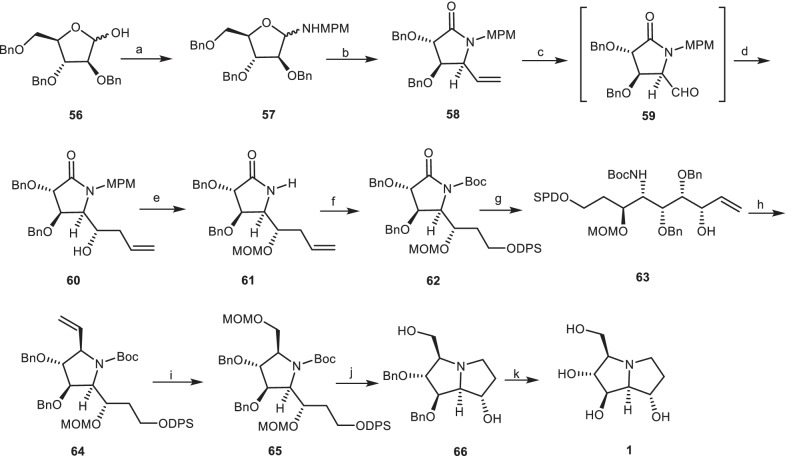
The synthesis of the alexine **1** by Yoda et al. Reagents and conditions: (a) MPMNH_2_, PhH/CHCl_3_ (1:1), reflux; (b) (i) CH_2_$=$CHMgBr, THF, − 78 to − 40 °C, 70%; (ii) PCC, CH_2_Cl_2_, 68%; (c) (i) OsO_4_, NMO, acetone/H_2_O (1:1), 98%; (ii) NaIO_4_, Et_2_O/H_2_O (2:1); (d) allyltrimethylsilane, BF_3_**·**OEt_2_, CH_2_Cl_2_, − 78 to − 20 °C, 82% (two steps); (e) (i) CAN, CH_3_CN/H_2_O (9:1), 71%; (ii) MOMCl, *i-*Pr_2_NEt, CH_2_Cl_2_, 75%; (f) (i) OsO_4_, NMO, acetone/H_2_O (1:1), 91%; (ii) NaIO_4_, Et_2_O/H_2_O (2:1); (iii) NaBH_4_, EtOH, 90%; (iv) DPSCl, imidazole, DMF; (v) (Boc)_2_O, DMAP, Et_3_N, CH_2_Cl_2_, 99%; (g) (i) CH_2_$=$CHMgBr, THF, − 78 °C; (ii) NaBH_4_/CeCl_3_, MeOH, − 45 °C, 66%; (h) (i) MsCl, Et_3_N, CH_2_Cl_2_; (ii) *t*-BuOK, THF, 84%; (i) (i) OsO_4_, NMO, acetone/H_2_O (1:1), 92%; (ii) NaIO_4_, Et_2_O/H_2_O (2:1); (iii) NaBH_4_, EtOH, 74%; (iv) MOMCl, *i-*Pr_2_NEt, CH_2_Cl_2_ 99%; (j) (i) Bu_4_NF, THF.; (ii) *p*-TsCl, pyridine, 92%; (iii) HCl, MeOH; (iv) K_2_CO_3_, MeOH, 94%; (k) H_2_, 10% Pd:C, EtOH, 70%.

Yoda et al*.* [32] reported the first asymmetric total synthesis of (+)-alexine **1** from the benzyl protected d-arabinofuranose derivative **56** as the chiral pool source and the functionalized homochiral lactam **58**. The compound **56** was protected with methoxyphenylmethyl amine (MPMNH_2_) to provide aminal **57**, which was reacted with vinylmagnesium bromide (CH_2_=CHMgBr) via nucleophilic addition, followed by oxidative reaction with pyridinium chlorochromate (PCC) to yield the functionalized homochiral lactam **58**. Dihydroxylation of the double bond of compound **58** provided the aldehyde intermediate **59**, which underwent BF_3_^**.**^OEt_2_-induced allylation to obtain the allyl alcohol **60** in two steps. Deprotection of *N*-MPM moiety of compound **60**, and then its OH group-protection with methoxymethyl chloride (MOMCl) provided compound **61**, which was submitted to oxidative cleavage of the terminal alkene, followed by reduction to the corresponding alcohol, and then protection by diphenylsilylchloride (DPSCl) and de-*tert*-butyldicarbonate [(Boc)_2_O] to provide the *N*-protected lactam **62**. Then, the addition of vinyl Grignard reagent to compound **62**, followed by reduction with NaBH_4_ afforded the compound **63**, which was cyclized under basic conditions after mesylation to give the pyrrolidine derivative **64**. Then, it was submitted to oxidative cleavage of alkene moiety, reduction, and protection reaction to provide the fully protected pyrrolidine derivative **65**, which was converted to the protected pyrrolizidine core **66** by the replacement of the silyl group with the Ts leaving group, and subsequent removing two MOM and Boc groups. Finally, deprotection of the benzyl groups of compound **66** gave the alexine **1** (Scheme 4).

**Scheme 5:**
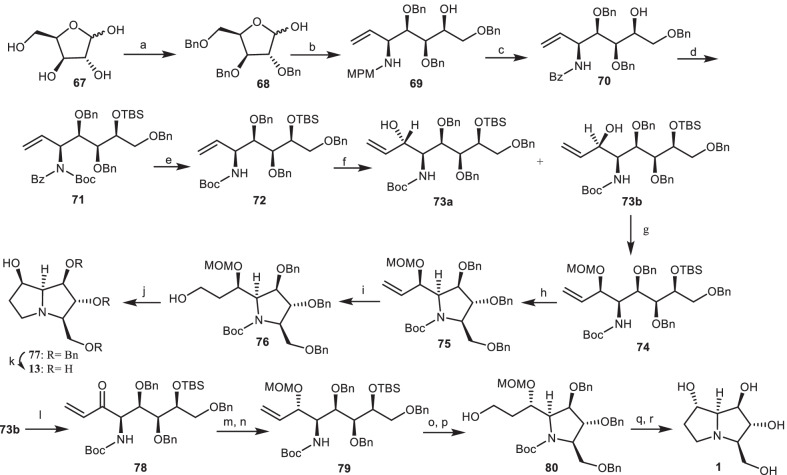
The synthesis of the alexine **1**, (−)-7-epi-alexine **13** by Yoda and his co-workers. Reagents and conditions: (a) ref [34]; (b) (i) MPMNH_2_, toluene, reflux; (ii) CH_2_=CHMgCl, THF, − 78 °C to 0 °C, 2 h, 75% (two steps); (c) (i) BzCl, CH_2_Cl_2_, 89%; (ii) CAN, MeOH, 86%; (d) (i) TBSCl, imidazole, DMF, 89%; (ii) Boc_2_O, Et_3_N, DMAP, CH_2_Cl_2_, 92%; (e) (Me_2_N) _2_C=NH, reflux, 130 °C, 8 h, 98%; (f) (i) OsO_4_, NMO, acetone, 94%; (ii) NaIO_4_, THF/H_2_O(1:1); (iii) CH_2_=CHMgCl, THF, − 78 °C, 95%; (g) MOMCl, *i*-Pr_2_NEt, 98%; (h) (i) Bu_4_NF, THF, 98%, (ii) MsCl, Et_3_N, CH_2_Cl_2_; (iii) *t*-BuOK, THF, 84%; (i) (i) 9-BBN, H_2_O_2_, NaOH, THF; 94%; (j) (i) MsCl, Et_3_N, CH_2_Cl_2_; (ii) BF_3_.OEt_2_, CH_2_Cl_2_, − 20 °C, 84%; (k) Pd/C (10%), HCO_2_NH_4_, MeOH, reflux, 2 h, 82%; (l) TPAP, NMO, CH_2_Cl_2_, 92%; (m) NaBH_4_, CeCl_3_, MeOH, − 45 °C; 80%; (n) MOMCl, *i*-Pr_2_NEt, 96%; (o) (i) Bu_4_NF, THF, 91%; (ii) MsCl, Et_3_N, CH_2_Cl_2_; (iii) *t*-BuOK, THF, 87%; (p) 9-BBN, H_2_O_2_, NaOH, THF, 97%; (q) (i) MsCl, Et_3_N, CH_2_Cl_2_; (ii) BF_3_.OEt_2_, CH_2_Cl_2_, − 20 °C, 84%; (r) Pd/C(10%), HCO_2_NH_4_, MeOH; reflux 2 h; 83%.

In another study, an efficient pathway for the asymmetric total synthesis of natural (+)-alexine **1** and (−)-7-epi-alexine **13** from protected l-xylose derivative **68** described by Yoda and his co-workers [33]. l-xylose **67** [34] was protected to provide the l-xylose derivative **68**, which was treated with 4-methoxybenzylamine (MPM amine), followed by nucleophilic addition of vinyl magnesium chloride to give the amino alcohol **69** as a single isomer. Through the treatment of compound **69** with benzoyl chloride, followed by the reaction with cerium ammonium nitrate (CAN), the MPM group was replaced with the *N*-benzoyl (Bz) protecting group to provide the amide **70** in two steps. Then, amide **70** was submitted to further protection reactions to obtain the *N-tert*-butyloxycarbonyl (*N*-Boc) amide **71**, which was deprotected to give the pure NHBoc carbamate **72**. The oxidative cleavage of olefinic moiety **72** afforded the aldehyde intermediate, which was treated with vinyl magnesium chloride (CH_2_=CHMgCl) to obtain allylic alcohol **73a** and **73b**. Due to the Cram’s non-chelation transition structure [35] the carbonyl group was attacked via the less hindered side, so compound **73b** is the main product. Its hydroxyl group was protected with methoxymethyl chloride (MOMCl) to give the compound **74**, which was subjected to the following reactions of desilylation, mesylation, and *t*-BuOK-promoted cyclization, leading to the pyrrolidine intermediate **75** in three steps. Hydroboration of the vinyl group of compound **75** gave the primary alcohol **76**, which was mesylated, followed by aminocyclization and accompanied by the removal of MOM and Boc group to provide the pyrrolizidine structure **77**. By hydrogenolysis of compound **77** with Pd/C, the deprotection of Bn groups was occurred to provide the desired alkaloid **13**. The synthesis of (+)-alexine **1** required the inversion in the configuration of the allylic hydroxy center of compound **73b**. For this purpose, oxidation of compound **73b** with *tetra-n*-propylammonium perruthenate (TPAP) provided the enone **78**, which was reduced diastereoselectively by NaBH_4_/CeCl_3_ (Luche reduction) [36], and followed by the protection of the alcohol group to yield allylic alcohol **79**. By using the same synthetic strategy to the synthesis of compound **13**, the synthesis of (+)-alexine **1** was completed (Scheme 5).

### The synthetic approach of Pearson and co-workers

**Scheme 6:**
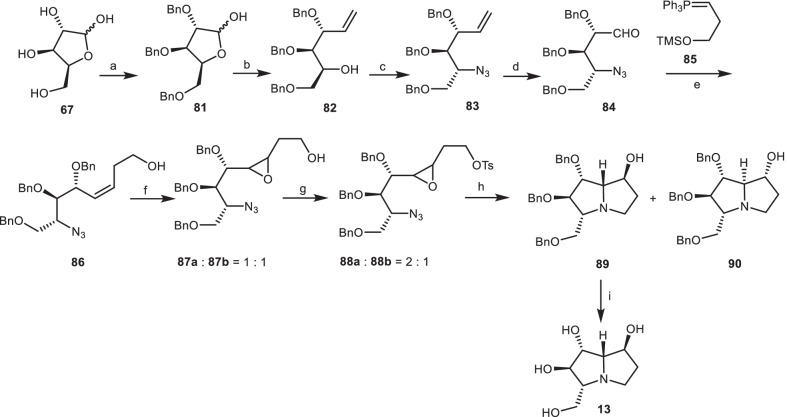
The synthesis of the (−)-7-epialexine **13** by Pearson et al. Reagents and conditions: (a) ref [39]; (b) Ph_3_PCH_3_Br, *n*-BuLi, THF, − 78 °C to r.t., 24 h, 66%; (c) (i) Tf_2_O, pyridine, CH_2_Cl_2_, − 40 °C to r.t., 3 h; (ii) *n*-Bu_4_NN_3_, PhH, 0 °C to r.t., 1 h, 75%; (d) (i) O_3_, MeOH, CH_2_Cl_2_, − 78 °C, 17 min; (ii) Me_2_S, − 78 °C to r.t., 2.25 h; (e) (i) **85**, KN(TMS)_2_, Me_3_SiCl, THF, -78 °C to r.t., 4 h; (ii) aq. HC1, 35%; (f) *m*-CPBA, CH_2_Cl_2_, 0 °C to r.t., 24 h, 65%; (g) TsCl, pyridine, CH_2_Cl_2_, − 15 °C, 48 h, 77%; (h) (i) H_2_, 10% Pd/C, ether/EtOH (2:1), r.t., 15 h; (ii) K_2_CO_3_, EtOH, reflux, 20 h, 71%; (i) H_2_, 10% Pd/C, EtOH, r.t., 48 h, 87%.

Pearson et al. [37, 38] reported the total synthesis of (−)-7-epialexine **13** by reductive amino-cyclization of azidoepoxide as a key step to yield the target compounds. Through the protection reaction, l-xylose **67** was converted to 2,3,5-*tri*-*O*-benzyl-l-xylofuranose **81** [39], which was transformed to the compound **82** via Wittig olefination. Then, it was converted to the azide **86** via replacement of resultant triflate with the azide group, which the latter was transformed to the aldehyde **84** by ozonolysis process, followed by the Wittig reaction with the silyloxy-substituted ylide **85** to yield the stereoselective *Z*-alkene **86**. It was treated with *m*-chloroperoxybenzoic acid (*m*-CPBA) to give epoxides **87a** and **87b** as a 1:1 mixture of non-separated isomers, which was tosylated to give compounds **88 a/b**, followed by the reduction of azido group, and then the aminocyclization of resultant amine to deliver a 2:1 mixture of pyrrolizidines **89**, and **90**. The target compound, (−)-7-epialexine **14** was produced via the deprotection of **89** using H_2_, Pd/C (Scheme 6).

### The synthetic approach of Wong and Romero

**Scheme 7:**
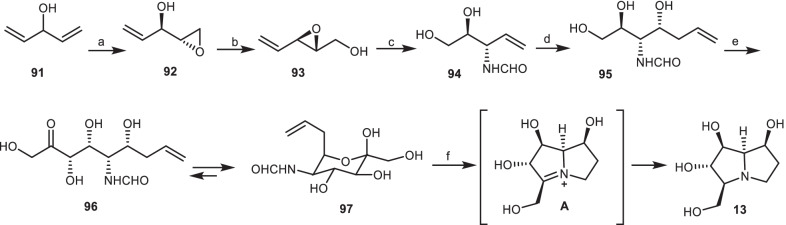
The synthesis of the 7-epialexine **13** by Wong and Romero. Reagents and conditions: (a) (−)-DIPT, C_6_H_5_C(CH_3_)_2_OOH, Ti(*i*PrO)_4_, CH_2_Cl_2_, − 35 °C, 36 h, 86%, > 99% ee; (b) NaOH, r.t., 20 min, 98%; (c) (i) NH_4_OH, r.t., 24 h; (ii) ethyl formate, EtOH, 90 °C, 18 h, 95%; (d) (i) O_3_, MeOH, − 78 °C; (ii) In, allyl bromide, H_2_O, r.t., 12 h, 56%; (e) (i) NaIO_4_, H_2_O; (ii) DHAP, FDPA, r.t., 72 h; (iii) Pase, 38 °C, 12 h, 25%; (f) (i) O_3_, MeOH/H_2_O, − 78 °C; (ii) H_2_, Pd/C, − 78 °C to r.t.,; (iii) HCl and H_2_, Pd/C, r.t., 72 h, 66%.

Wong and Romero [40], reported the first chemo-enzymatic approach to the synthesis of 7-epialexine **13** without any protecting the group. In the first step, through the Sharpless asymmetric epoxidation, divinylcarbinol **91** was converted to epoxide **92**, which was undergoing base-induced rearrangement to give the epoxide **93**. Ring-opening of epoxide **93** using ammonia, followed by the protection of resultant amine with ethyl formate provided the formamide **94**. Through the ozonolysis, it converted to the mixture of hemiacetals, which were transformed to triol **95** in the presence of allyl bromide and indium. The terminal diol of **95** submitted to NaIO_4_ cleavage to obtain the corresponding aldehyde, which underwent to aldol reaction with dihydroxyacetone phosphate (DHAP) mediated by fructose-1,6-diphosphate aldolase (FDPA), followed by the enzymatic cleavage of the phosphate group using acid phosphatase (Pase) to yield tetrol **96**, which was in equilibrium with pyranose **97**. Ozonolysis of compound **97** under the reductive conditions using H_2_, Pd/C, followed by acidic cleavage of the formamide, and subsequent reduction of intermediate **A** provided the target compound 7-epialexine **13** (Scheme 7).

### The synthetic approach of Donohoe and co-workers

**Scheme 8:**
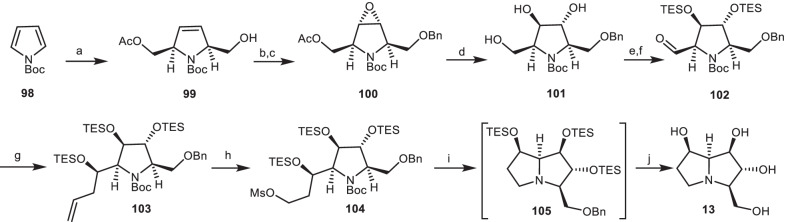
The synthesis of the (−)-7-epi-alexine **13** by Donohoe’s group. Reagents and conditions: (a) ref [42]; (b) BnBr, Ag_2_O, CH_2_Cl_2_, r.t., 12 h, 73%; (c) KHSO_5_, CF_3_COCH_3_, EDTA, aq. MeCN, 0 °C, 30 min, 96% (98%ee); (d) (i) BF_3_.OEt_2_, CH_2_Cl_2_, − 50 °C, 14 h; (ii) K_2_CO_3_, MeOH, r.t., 2 h, 73%; (e) Et_3_SiCl, DMAP, Et_3_N, CH_2_Cl_2_, r.t., 16 h, 84%; (f) (COCl)_2_, DMSO, Et_3_N, CH_2_Cl_2_, − 78 °C, 1 h, 76%; (g) (i) Allyl magnesium bromide, THF, − 78 °C, 1 h; (ii) Et_3_SiCl, DMAP, Et_3_N, CH_2_Cl_2_, r.t., 16 h, 86% (6:1 dr); (h) (i) O_3_, Ph_3_P, CH_2_Cl_2_, r.t., 12 h, (ii) NaBH_4_, CH_2_Cl/MeOH, 0 °C, 10 min,; (iii) Ms_2_O, 2,6-lutidine, DMAP, CH_2_Cl_2_, r.t., 12 h, 74%; (i) TESOTf, 2,6-lutidine, CH_2_Cl_2_, then MeOH, r.t., 12 h; (j) H_2_, Pd/C, HC1, MeOH, r.t., 89%.

Since the target molecule **13** had the *trans*-3,4-diol stereochemistry, the *trans*-3,4-diol **101** had to be obtained to enable the synthesis of compound **13**. Donohoe’s group utilizes *N*-Boc pyrrole **98** to provide monoacetate **99** [41, 42]. Then, it was submitted to benzyl protection, followed by epoxidation to provide the epoxide derivative **100**. Through the ring-opening reaction of epoxide **100**, and subsequent deprotection of acetate group under basic conditions gave triol **101**. The protection and oxidation of compound **101** under Swern conditions gave aldehyde **102**, which was reacted with allyl magnesium bromide to generate the protected secondary alcohol **103**. The double bond of compound **103** was cleaved and reduced to the primary hydroxyl group, followed by mesylation with Ms_2_O to produce **104**. Then, it was deprotected and cyclized to obtain pyrrolizidine **105**. Removal of protecting groups of compound **105** under acidic hydrogenolysis conditions afforded the (−)-7-epialexine **13** (Scheme 8).

### The synthetic approach of Han and co-workers

**Scheme 9:**
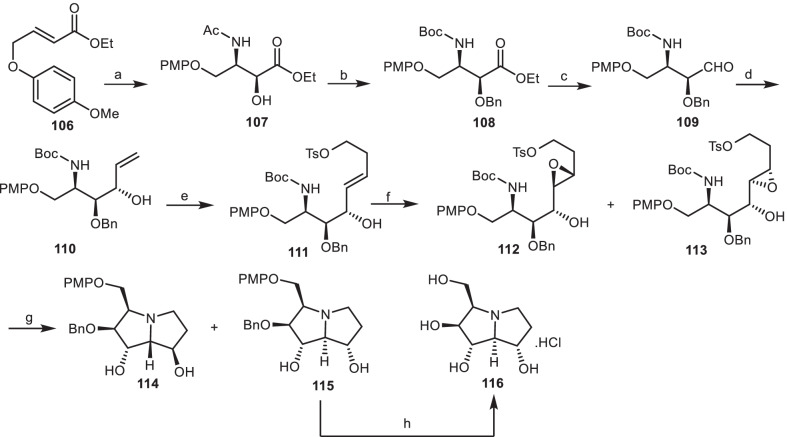
The synthesis of the 1,2-diepialexine **116** by Han and his co-workers. Reagents and conditions: (a) K_2_OsO_4_.2H_2_O, (DHQD)_2_-PHAL, LiOH, CH_3_CONHBr, *t*-BuOH/H_2_O (2:1), 4 °C, 8 h, 70%; (b) (i) NaH, BnCl, DMF, 0 °C, 10 h, 78%; (ii) (Boc)_2_O, DMAP, THF, reflux, 4 h, then NH_2_NH_2_, MeOH, 4 h, 85%; (c) DIBALH, CH_2_Cl_2_, − 78 °C, 3 h, 90%; (d) CH_2_=CHMgBr, THF, − 50 °C, 1 h, then r.t., 1 h, 85%; (e) Grubbs’ catalyst, C_11_H_14_O_3_S, CH_2_Cl_2_, 65%; (f) VO(acac)_2_, TBHP, toluene, 74%; (g) (i) HCl, MeOH, (ii) K_2_CO_3_, MeOH, 83%; (h) (i) CAN, MeCN/H_2_O (4:1), 4 °C; (ii) H_2_, Pd/C, MeOH, **116** (72%).

Han and his co-workers [43], reported the first asymmetric synthesis of 1,2-diepialexine **117** from the simple olefin **106** in 10 steps (Scheme 9). The regioselective asymmetric aminohydroxylation of *α,β*-unsaturated ester **106** afforded the enantioselective *syn*-amino alcohol **107**, followed by the OH protection with BnCl, and the *N*-acetyl group was transformed to the *N*-Boc group to provide the carbamate **108**, which was reduced to the aldehyde **109**. Then, it was reacted with vinyl magnesium bromide to provide the allylic alcohol **110**, which was undergone an olefin cross-metathesis [44] with 4-butenol *p*-tolylsulfonate (C_11_H_14_O_3_S) to yield the *trans*-olefin **111**. It was subjected to VO(acac)_2_ and produced the mixture of the epoxides **112** and **113**, which were treated with HCl, and then by K_2_CO_3_ to generate the double cyclized compounds **114** and **115**. 1,2-Diepialexine **116** was produced as a HCl salt, by deprotection of *p*-methoxybenzyl (PMP) and benzyl group by ceric ammonium nitrate (CAN), and then hydrogenation of compound **115**.

### The synthetic approach of Somfai and co-workers

**Scheme 10:**
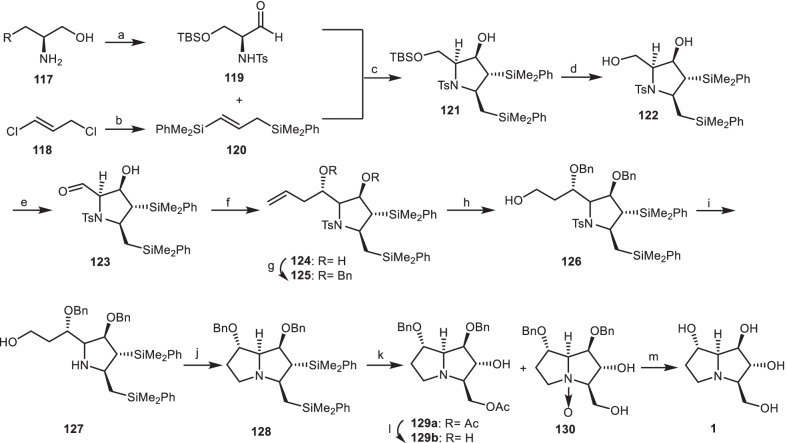
The synthesis of the alexine **1** by Somfai et al. Reagents and conditions: (a) ref [46]; (b) ref [47]; (c) MeAlCl_2_, CH_2_Cl_2_, −78 °C, 57% (> 40:1 dr); (d) AcOH, THF/H_2_O, r.t., 96%; (e) TEMPO, NaOCl, KBr, CH_2_Cl_2_/H_2_O, 0 °C, 99%; (f) CH_2_=CHCH_2_TMS, TiCl_4_, CH_2_Cl_2_, − 78 °C, 1.5 h, 70%; (g) BnBr, KHMDS, THF, − 78 °C, 85%; (h) (i) OsO_4_, NaIO_4_, Py, CH_3_CN/H_2_O, r.t., (ii) NaBH_4_, MeOH, 0 °C, 72%; (i) NaC_10_H_8_, DME, − 60 °C, 99%; (j) CBr_4_, Ph_3_P, Et_3_N, CH_2_Cl_2_, r.t, 64%; (k) Hg(OTf)_2_, AcO_2_H, AcOH, r.t., **129a** (41%), **129** (21%); (l) LiOH, THF/H_2_O, r.t., 90%; (m) H_2_, Pd/C, EtOH, r.t., 70%.

A total asymmetric synthesis of natural alkaloid alexine **1** based on stereoselective [3 + 2] annulation reaction of chiral *α*-amino aldehyde **119** with 1,3-bis(silyl)propene **120** was introduced by Somfai et al. [45]. Aldehyde **119** (obtained from *α*-amino alcohols **117**) [46] was reacted with silane **120** (obtained from 1,3-dichloropropene **118**) in the presence of (CH_3_)_3_SiCl) [47] to afford pyrrolidine **121**, through the stereoselective [3 + 2] annulation reaction. Removal of the silyl group of **121** gave diol **122**, which was oxidized chemoselectivity to give aldehyde **123**. Then, it was reacted with allyltrimethylsilane (CH_2_=CHCH_2_TMS) and TiCl_4_ to obtain pyrrolidine **124**, followed by the protection of OH group to yield the compound **125**. The terminal olefin moiety of compound **125** was cleavaged by Lemieux-Johnson oxidation [48] to afford the corresponding aldehyde, which was reduced to alcohol **126** in two steps.The tosyl (Ts) group of compound **126** was removed to provide amine **127**. Its hydroxyl group was converted into the corresponding alkylbromide, which was cyclized to the pyrrolizidine **128**. Chemoselective oxidation of its silyl group under Tamao-Fleming condition provided pyrrolizidine **129a** and *N*-oxide derivative **130**. The acetate group of compound **129a** was hydrolyzed to provide the alcohol **129b**. The hydrogenolysis of compound **129b** and **130** with H_2_, Pd/C, gave alexine **1** (Scheme 10).

**Scheme 11:**
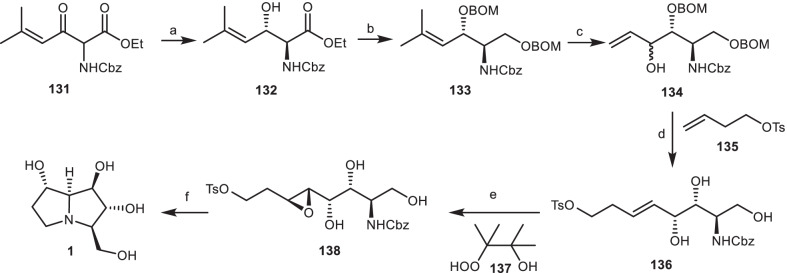
The synthesis of the alexine **1** by Somfai et al. **Reagents and conditions**: (a) [RuCl_2_(mesitylene)]_2_, (*S,S*)-DPAE, HCO_2_H/EtN_3_, dioxane, r.t., 77%; (b) (i) NaBH_4_, EtOH, r.t.; (ii) BOMCl, *i*-PrNEt_2_, CHCl_3_, r.t., 78%; (c) (i) O_3_/DMS, CH_2_Cl_2_, − 78 °C to r.t.; (ii) ZnCl_2_, CH_2_=CHMgBr, CH_2_Cl/CH_3_Ph, − 78 °C to r.t., 58%; (d) (i) Grubbs’ catalyst, CH_2_Cl_2_, r.t.; (ii) HCI, MeOH, 0 °C to r.t., 67%; (e) Ti(OiPr)_4_, CH_2_Cl_2_, 78% (f) H_2_, Pd/C, MeOH, 76%.

In another work, Somfai and his co-workers [49], developed a new approach based on asymmetric synthesis of the alkaloid alexine **1** through Dynamic Kinetic Resolution/Asymmetric Transfer Hydrogenation Reaction (DKR/ATH) [50]. *γ*-Alkenyl-*β*-keto-*α*-amido esters **131** was subjected to [RuCl_2_(mesitylene)]_2_, (*S,S*)-DPAE, HCO_2_H/EtN_3_ through the ATH reaction, using the optimized conditions, furnished diastereo- and enantioselective anti-*α*-amido-*β*-hydroxy ester **132** in high yield. Then, it was reduced and protected to give compound **133**, which was ozonized, followed by the addition of vinyl magnesium bromide to afford the compound **134**. It was reacted with compound **135** via the cross-metathesis reaction, followed by the deprotection of benzyloxymethyl (BOM) group to provide compound **136**. Then, it was treated with *β*-hydroperoxy alcohol **137** through the neighboring group-directed epoxidation in the presence of Ti(OiPr) _4_ to give anti epoxide **138**, which was deprotected to afford alexine **1** (Scheme 11).

### The synthetic approach of Davies and co-workers

**Scheme 12:**
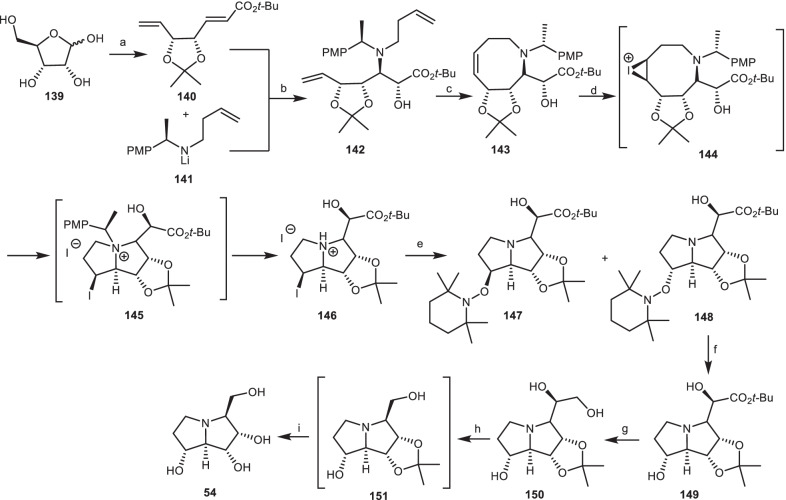
The synthesis of the 1-epialexine **54** by Davis et al. Reagents and conditions: (a) ref [52]; (b) (i) THF, − 78 °C, 2 h; (ii) (−)-CSO, − 78 °C to r.t., 12 h, 50% (> 99:1 dr); (c) (i) Grubbs’ catalyst, CH_2_Cl_2_, 30 °C, 12 h; (ii) tri(hydroxymethyl) phosphine, Et_3_N, r.t., 12 h, 73%; (d) I_2_, NaHCO_3_, CH_2_Cl_2_, r.t., 12 h, 79%; (e) Bu_3_SnH, TEMPO, PhMe, 70 °C, 1.5 h, **147** (19%), **148** (69%), (> 99: 1 dr); (f) Zn, AcOH, THF, H_2_O, 70 °C, 2 h; (g) LiAlH_4_, THF, − 78 °C to r.t., 12 h; (h) NaIO_4_, MeOH, H_2_O, r.t., 4 h then NaBH_4_, r.t., 12 h; (i) HC1 (3.0 M aq.), MeOH, reflux, 2 h.

Davis et al. [51] developed an efficient asymmetric synthesis of 1-epialexine **54**, based on transannular iodoamination as a critical step. Lithium (*R*)-*N*-but-3-enyl-*N*-(*α*-methyl-*p*-methoxybenzyl) amide **141** was reacted with unsaturated ester **140** (derived from *d*-ribose **139**) [52] in the presence of (−)-camphorsulfonyloxaziridine [(−)-CSO] to obtain amino alcohol **142**, through the conjugate addition reaction and enolate oxidation. Subsequent treatment of amino alcohol **142** with Grubbs’ catalyst gave hexahydroazocine **143**, which was reacted with I_2_, NaHCO_3_, CH_2_Cl_2_ by the diastereoselective transannular iodoamination to give the hydroiodide salt **146**, as demonstrated in (Scheme 12). Treatment of compound **146** through the radical-mediated addition of tributyltin hydride (Bu_3_SnH) in the presence of tetramethylpiperidinyl-*N*-oxyl (TEMPO) provided a mixture of diastereoisomeric pyrrolizidines **147** and **148**. Cleavage of the N–O bond in pyrrolizidine **148** in the presence of Zn, AcOH gave the corresponding hydroxypyrrolizidine **149**, which was converted to the triol **319** by reduction of carboxylate group. Oxidative cleavage of triol **150** with NaIO_4_, followed by reduction with NaBH_4_ gave the pyrrolizidine alcohol **151**. Hydrolysis of acetonide group, through the reaction of pyrrolizidine alcohol **151** with HCl, led to (−)-1-epialexine **54**.

### The synthetic approach of Myeong and Ham

**Scheme 13:**
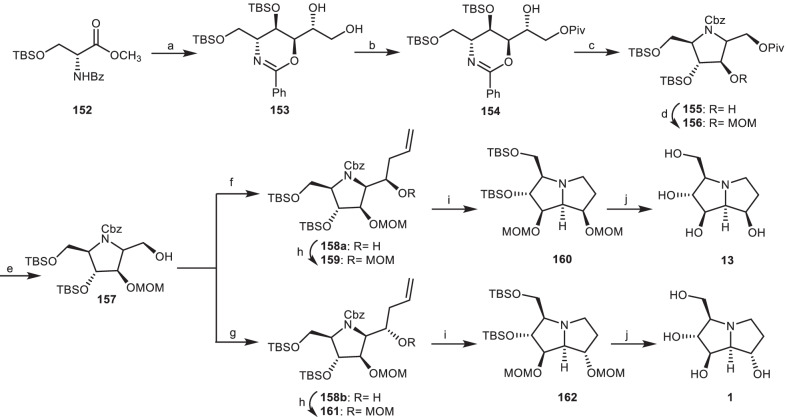
The synthesis of the (−)-7-epi-alexine **13** and alexine **1** by Myeong and Ham. Reagents and conditions: (a) ref [54]; (b) PivCl, pyridine, CH_2_Cl_2_, 0 °C, 1 h, 91%; (c) (i) MsCl, Et_3_N, CH_2_Cl_2_, 0 °C, 1 h, 93%; (ii) Pd(OH)_2_/C, H_2_ (80 psi), r.t., 24 h; (iii) CbzCl, Et_3_N, CH_2_Cl_2_, r.t., 1 h, 66%; (d) MOMCl, DIPEA, DMAP, CH_2_Cl_2_, 40 °C, 24 h, 83%; (e) DIBALH, CH_2_Cl_2_, − 78 °C, 1 h, 85%; (f) MgBr_2_.OEt_2_, AllylSnBu_3_, CH_2_Cl_2_, 3 h, 78%; (g) BF_3_.OEt_2_, AllylSnBu_3_, CH_2_Cl_2_, 3 h, 73%; (h) MOMCl, DIPEA, DMAP, CH_2_Cl_2_, 40 °C, 24 h, 83–85%; (i) (i) O_3_, MeOH, −78 °C, then Me_2_S; (ii) Pd(OH)_2_/C, H_2_, r.t., 24 h, 66–69%; (j) 1 N aq HCl, MeOH, 40 °C, 3 h, then DOWEX 50X-8 200, 87–90%.

In another study, Myeong and Ham achieved the total synthesis of alexine **1** and (−)-7-epi-alexine **13** from *anti,syn,anti*-oxazine **153** [53]. Treatment of diol **153** (obtained from **152**) [54] with 2,2-dimethylpropanoyl chloride (PivCl) gave compound **154**, which was mesylated, followed by oxazine ring cleavage, and simultaneously induced cyclization to afford functionalized pyrrolidine **155**. Protection of secondary alcohol **155** by a methoxymethyl (MOM) group gave the compound **156**, which was deprotected to give primary alcohol **157**. Stereoselective allylation of primary alcohol **157** using MgBr_2_.OEt_2_ and BF_3_.OEt_2_ resulted in the *syn*-alcohol **158a** and *anti*-alcohol **158b**, which were protected to give compounds **159** and **161**. Through the ozonolysis and hydrogenolysis process, they were converted to pyrrolizidine compounds **160** and **162**, which were reacted with HCl, followed by neutralization by an ion-exchange resin to provide (−)-7-epi-alexine **13** and alexine **1**, respectively (Scheme 13).

## Conclusion

Pyrrolizidine alkaloids called alexine **1** being discovered for the first time in 1988. The diverse array of potentially useful biological activities, as well as richness of stereochemical behavior of these alkaloids, have made these compounds attractive and valuable synthetic targets. In the present review, it was tried to highlight the varieties of strategies and methodologies towards the total synthesis of natural alexine **1**, and its isomers. It was expected much further development of this type of compounds in synthetic chemistry due to their structural features and biological activities.

## Abbreviations

ATH: Asymmetric transfer hydrogenation
Boc: *tert*-Butyloxycarbonyl
BOM: Benzyloxymethyl
CAN: Ceric ammonium nitrate
Cbz: Carbobenzyloxy
*m*-CPBA: *meta*-Chloroperoxybenzoic acid
CSO: Camphorsulfonyloxaziridine
DHAP: Dihydroxyacetone phosphate
(DHQD)_2_-AQN: 1,4-Bis(dihydroquinidinyl)anthraquinone
DHQDPHN: Dihydroquinidine-9-*O*-(9′-phenanthryl) ether
DIBALH: Diisobutylaluminium hydride
DIPT: Diisopropyl tartrate
DKR: Dynamic kinetic resolution
DPAE: 2-Amino-1,2-diphenyl-ethanol
DPSCl: Diphenylsilylchloride
DMAP: 4-Dimethylaminopyridine
DMS: Dimethylsulfide
EDTA: Ethylenediaminetetraacetic acid
FDPA: Fructose-1,6-diphosphate aldolase
KHMDS: Potassium bis(trimethylsilyl)amide
MAPh: Magnesium ammonium phosphate hexahydrate
MOM: Methoxymethyl
MPM: Methoxyphenylmethyl
NMO: *N*-Methylmorpholine-*N*-oxide
PCC: Pyridinium chlorochromate
PivCl: Dimethylpropanoyl chloride
TBAF: Tetra-*n*-butylammonium fluoride
TBDPS: *tert*-Butyldiphenylsilyl
TBHP: *t*-Butylhydroperoxide
TBS: *tert*-Butyldimethylsilyl
TBSOTf: *tert*-Butyldimethylsilyl trifluoromethanesulfonate
TEMPO: Tetramethylpiperidinyl-*N*-oxyl
TES: Triethylsilyl
TESOTf: Triethylsilyl trifluoromethanesulfonate
TFA: Trifuoroacetic acid
THF: Tetrahydrofuran
TMS: Trimethylsilyl
TPAP: Tetra-*n*-propylammonium perruthenate
